# Inhibitory Properties of Cysteine Protease Pro-Peptides from Barley Confer Resistance to Spider Mite Feeding

**DOI:** 10.1371/journal.pone.0128323

**Published:** 2015-06-03

**Authors:** M. Estrella Santamaria, Ana Arnaiz, Mercedes Diaz-Mendoza, Manuel Martinez, Isabel Diaz

**Affiliations:** Centro de Biotecnologia y Genomica de Plantas, Universidad Politecnica de Madrid, Autovia M40 (km 38), Pozuelo de Alarcon, 28223 Madrid, Spain; Ghent University, BELGIUM

## Abstract

C1A plant cysteine proteases are synthesized as pre-pro-enzymes that need to be processed to become active by the pro-peptide claves off from its cognate enzyme. These pro-sequences play multifunctional roles including the capacity to specifically inhibit their own as well as other C1A protease activities from diverse origin. In this study, it is analysed the potential role of C1A pro-regions from barley as regulators of cysteine proteases in target phytophagous arthropods (coleopteran and acari). The *in vitro* inhibitory action of these pro-sequences, purified as recombinant proteins, is demonstrated. Moreover, transgenic Arabidopsis plants expressing different fragments of *HvPap-1* barley gene containing the pro-peptide sequence were generated and the acaricide function was confirmed by bioassays conducted with the two-spotted spider mite *Tetranychus urticae*. Feeding trials resulted in a significant reduction of leaf damage in the transgenic lines expressing the pro-peptide in comparison to non-transformed control and strongly correlated with an increase in mite mortality. Additionally, the analysis of the expression levels of a selection of potential mite targets (proteases and protease inhibitors) revealed a mite strategy to counteract the inhibitory activity produced by the C1A barley pro-prodomain. These findings demonstrate that pro-peptides can control mite pests and could be applied as defence proteins in biotechnological systems.

## Introduction

Among 20–30,000 genes encoded by a plant genome, almost one thousand correspond to proteases, and more than one hundred belong to the 15 known families of cysteine-proteases (CysProt) [1]. The papain C1A family is the most abundant and its members are divided in cathepsin L-, B-, H- and F-like subgroups [2]. C1A protease group is extensively present in land plants, with members ranking from 32 in Arabidopsis to 45 in rice. In barley (*Hordeum vulgare*), the whole CysProt C1A family encoding 41 non-redundant genes has been identified and its participation in different physiological processes has been reported [3–6]. Individual C1A protease members are involved in a variety of proteolytic and physiological processes in plants such as senescence, abscission, programmed cell death, fruit ripening, pollen development, and the mobilization of proteins accumulated in seeds and tubers [6–9]. Besides, their implication in local and systemic defense responses against pathogens and pests has been also published [10–13]. All C1A proteins contain several disulphide bonds and share a conserved catalytic triad formed by a cysteine, a histidine and an asparagine. C1A peptidases from plants are synthesized as pre-proenzymes in the lumen of the endoplasmic reticulum (ER), where the signal peptide is removed. Then, via the trans-Golgi network they are transported to lytic vacuoles, senescent-associated vacuoles and lysosomes, or externally secreted [3, 14–16]. Alternatively, C1A protease precursors are stored in ER-derived organelles (endosperm-cotyledon-embryo ricinosomes and protein bodies) from where they are released upon acidification of the cytoplasm [5, 17, 18]. The relatively acidic pH of these compartments provides the optimal conditions for protease processing and activation by removing of the N-terminal pro-peptide.

The C1A N-terminal pro-peptides, of 130–160 amino acids long, are involved in the inhibition of their cognate enzyme, participate in the correct subcellular location of the protease and assist in folding of the mature enzyme [19, 20]. The pro-peptides contain the non-contiguous ERFNIN signature found in cathepsin L- and H-like or the ERFNAQ variant in cathepsin F-like, while cathepsin B-like proteases lack this motif [7, 12]. Interestingly, C1A pro-peptides from different species, including plants, have the capacity to inhibit several exogenous C1A peptidases [21–23]. Molecular modelling of three-dimensional protein structures has shown that most of the specific inhibitory properties of barley pro-peptides are determined from their interactions with the mature proteases [23]. *In vitro* assays and artificial diets using the recombinant pro-region of the papaya proteinase IV have demonstrated a selective inhibition of digestive CysProt from several phytophagous beetles [24]. Similarly, pro-regions from two plant pest species, the nematode *Heterodera glycines* and the bean bruchid *Acanthoscelides obtectus*, efficiently inhibited their own CysProt and proteases from other herbivory species [25, 26]. Moreover, it has been demonstrated that the CysProt pro-region from *H*. *glycines* expressed in soybean roots conferred protection against the cyst nematode infestation [27]. In this context, the development of protease inhibitors with strong specific inhibitory effects to the targeted organism represents a worthwhile but challenging task. The specificity of the pro-peptide inhibition is a crucial feature to be applied as regulators of CysProt in biotechnological systems. Therefore, plant protease pro-regions can be powerful tools for pathogen and pest control acting in similar way than the specific inhibitors of CysProt known as cystatins [12, 28–30].

Phytophagous insect and acari rely on digestive proteases, carbohydrases and lipases to become macromolecules in absorbable end-products. The pH and redox potential of insect/acari guts determine the optimal conditions for enzyme activity and the quality and quantity of nutrients that can be digested. Among arthropod pests the gut pH is in the slightly acidic to neutral range, with the exception of the alkaline midgut of lepidopteran or the acidic specific regions of midgut of hemipteran and dipteran [31]. Accordingly, the proteolytic activity profile in phytophagous coleopteran and acari species have shown the presence of CysProt as the most important digestive enzymes in their guts with a 5.0–7.0 pH range [32–34]. Focusing on phytophagous acari, the polyphagous two-spotted spider mite *Tetranychus urticae* (Acari: Tetranychidae) is one of the most devastating agriculture pest worldwide. It feeds on more than 150 crop species, including a wide range of greenhouse and annual and perennial field cultivars [35]. The fight against *T*. *urticae* is affected by: its quick development of pesticide resistance due to its short generation time and high population rate [36]; the scarce existence of resistant plant cultivars, and their resistance to Bt toxins expressed in transgenic plants [37]. The recent annotation of the spider mite genome has allowed identifying a large proliferation of gene families associated with digestion and detoxification of plant secondary compounds [38]. The proteolytic digestion of *T*. *urticae* is based mostly on CysProt activities, which is consistent with the strong proliferation of the C1A papain and C13 legumain CysProt gene families found in its genomic sequence [39].

In this study, it is analyzed the *in vitro* insecticidal/acaricide capabilities of CysProt pro-regions purified as recombinant proteins against important phytophagous coleopteran and acari. Likewise, *Arabidopsis thaliana* plants expressing different CysProt regions of HvPap-1 are generated to test their potential *in vivo* protection against *T*. *urticae*.

## Results

### Inhibitory properties of recombinant CysProt pro-peptides against proteolytic activities of different arthropods

**Fig 1:**
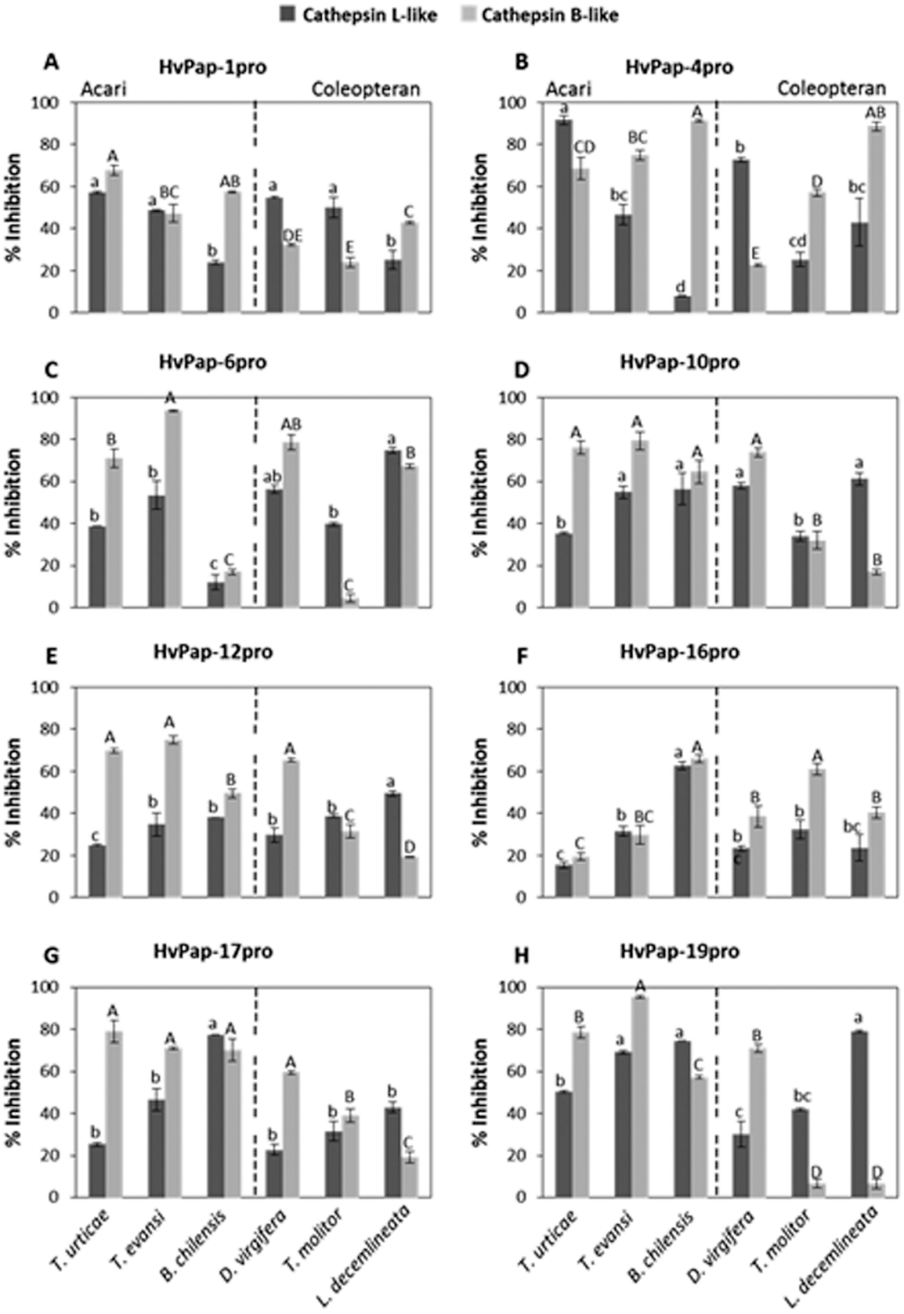
Inhibition of CysProt activities of arthropod protein extracts by barley C1A pro-peptides. Inhibitory activity of eight recombinant pro-peptides from barley C1A CysProt (HvPap-1pro, HvPap-4pro, HvPap-6pro, HvPap-10pro, HvPap-12pro, HvPap-16pro, HvPap-17pro and HvPap-19pro) against crude protein extracts of phytophagous acari (*T*. *urticae*, *T*. *evansi* and *B*. *chilensis*) and phytophagous coleopteran (*L*. *decemlineata*, *T*. *molitor* and *D*. *virgifera)*. Specific substrates of cathepsin L- and B-like were used. Data are mean ± SE of triplicate measurements of each sample. Values are expressed as % of inhibition. Different letters indicate significant differences (P<0.05, HSD test).

The inhibitory capability of eight recombinant pro-peptides derived from different barley C1A CysProt (HvPap-1pro, HvPap-4pro, HvPap-6pro, HvPap-10pro, HvPap-12pro, HvPap-16pro, HvPap-17pro and HvPap-19pro) was *in vitro* tested against crude protein extracts of three phytophagous coleopteran species (*Leptinotarsa decemlineata*, *Diabrotica virgifera* and *Tenebrio molitor*) and three phytophagous acari species (*T*. *urticae*, *T*. *evansi and Brevipalpus chilensis*). Previous alignment of the pro-peptide sequences showed strong differences in the amino acid residues (S1 Fig). Specific substrates susceptible to be degraded by cathepsin L- and B-like were used for all samples. The inhibition profiles showed that all recombinant pro-peptides reduced cathepsin L- and B-like activities of both insects and mites, although differences among pro-peptides and the target arthropods were observed (Fig 1). In general, cathepsin B-like activity detected in the crude extracts from the three spider mites was more susceptible to be inhibited by the pro-peptides than the cathepsin L-like activity. A wider variability was found when the *in vitro* inhibitory assays were performed with protein extracts from coleopteran species. The pro-peptide HvPap-19pro reduced about 80% of *L*. *decemlineata* cathepsin L-like activity, while same inhibitory levels of cathepsin B-like action were produced by the pro-peptide HvPap-4pro in the same arthropod species. Similarly, cathepsin B-like proteolytic patterns of *D*. *virgifera* were highly inhibited by HvPap-6pro, HvPap-10pro HvPap-12pro and HvPap-19pro whereas HvPap-4pro was the strongest inhibitor of cathepsin L-like activity of this coleopteran. The CysProt proteolytic activities of *T*. *molitor* extracts were less susceptible to be blocked by the pro-peptides and percentages of protease activity inhibition were not higher than 65%.

### Molecular characterization of Arabidopsis plants expressing different *HvPap-1* gene fragments from barley

**Fig 2:**
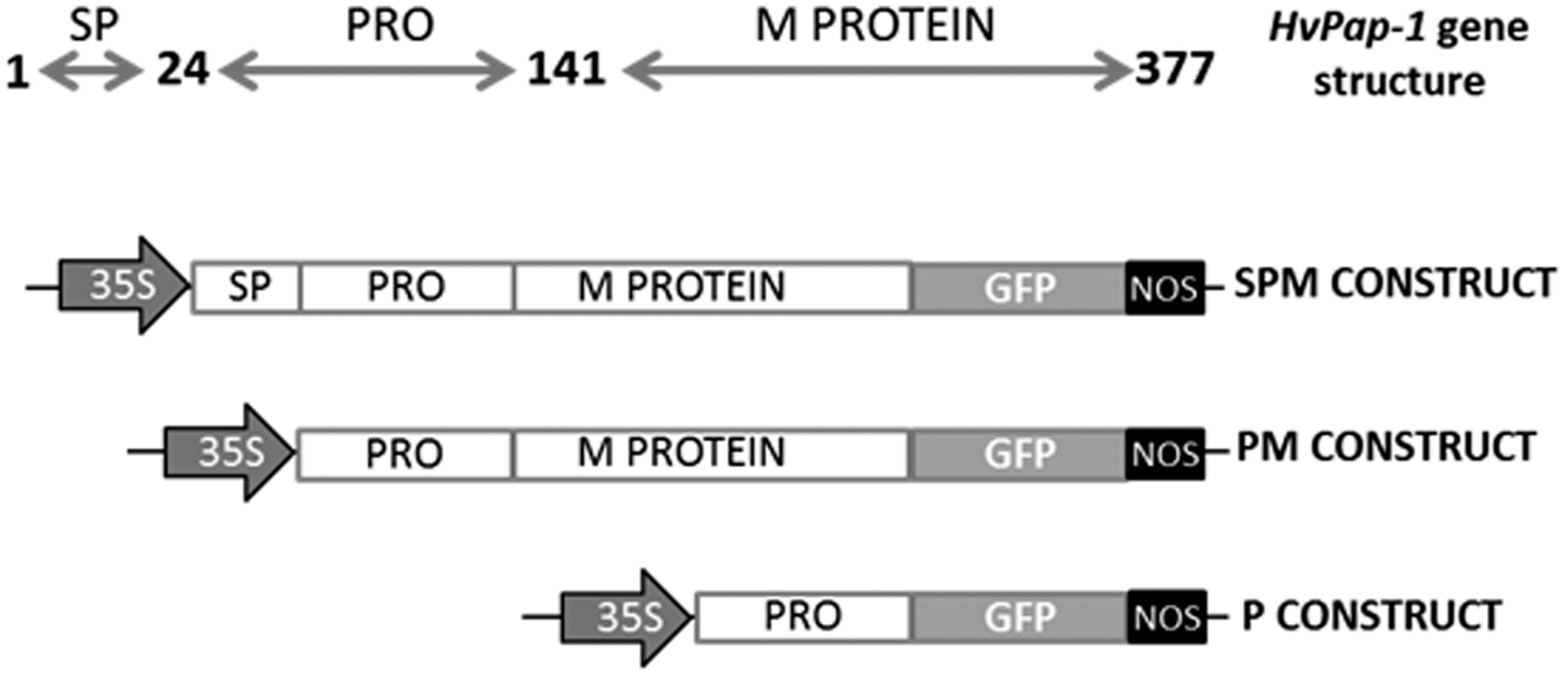
Scheme of *HvPap-1* gene structure and the derived gene constructs. SP: signal peptide; PRO: pro-peptide; M PROTEIN: mature protein. Numbers indicate amino acid positions. Constructs (SPM, PM and P) containing different fragments of the *HvPap-1* gene under the CaMV35S promoter were fused to the *GFP* gene followed by the NOS terminator.

Transgenic Arabidopsis lines were generated in hygromycin medium after Agrotransformation of three independent constructs (SPM, PM and P) containing different fragments derived from the barley *HvPap-1* gene (Fig 2). T2 generation was recovered and screened for the presence of the transgenes by genomic conventional PCR. Independent plants of each transgenic line derived from each construct (SPM: 1.1, 1.2 and 1.3 lines; PM: 2.1, 2.2. and 2.3 lines and P: 3.1, 3.2 and 3.3 lines) exhibited the expected amplified bands of 1131, 1059 and 351 bp, respectively, which were absent in the non-transformed plants (S2 Fig). No phenotypic differences were observed in transformed lines in comparison to the Col control plants.

**Fig 3:**
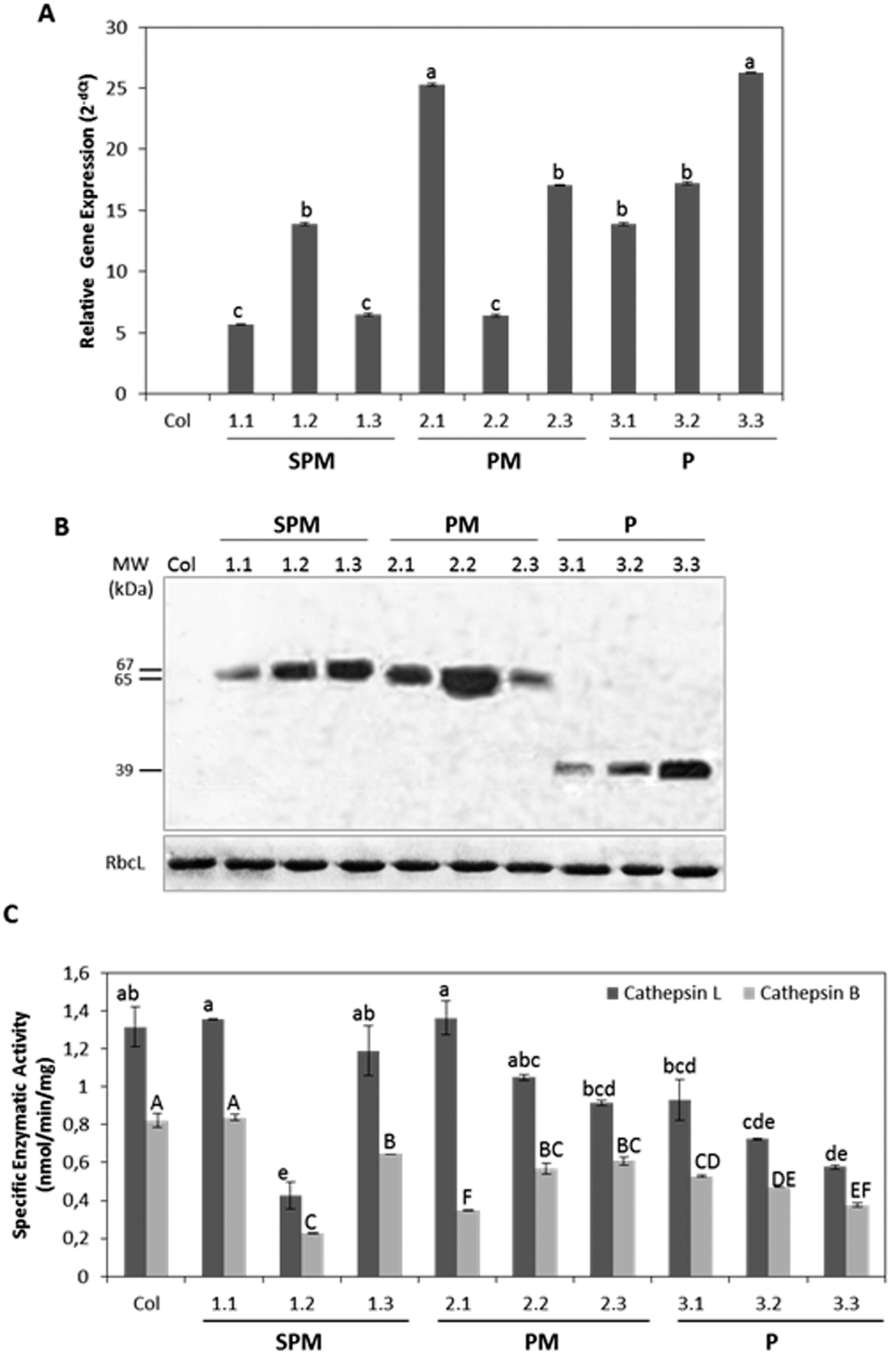
Analysis of the mRNA, protein expression and proteolytic activities of the transformed and control Arabidopsis plants. A. Analysis of the mRNA expression levels of T2 transformed lines and non-transformed control (Col) by RTq-PCR. Values expressed as the relative mRNA levels of the different transgenes were normalized to the Arabidopsis ubiquitin gene expression. B. Protein content detected by western-blot analysis of T2 transformed lines and non-transformed control (Col), using 10 μg of protein/lane and anti-GFP and anti-Rubisco antibodies. Large subunit of Rubisco (RbcL) was used as control loading. MW: molecular weights are indicated in kDa. C. Proteolytic activities of T2 transformed lines and non-transformed control (Col) expressed as nmoles/min/mg, using specific substrates of cathespin L- and B-like. Data are mean ± SE of triplicate measurements of each sample. Different letters indicate significant differences (P<0.05, Tukey's HSD). Transgenic plants were: SPM (lines 1.1, 1.2, 1.3), PM (lines 2.1, 2.2, 2.3) and P (lines 3.1, 3.3, 3.3).

The expression of the entire *HvPap-1* gene, the *HvPap-1* gene without signal peptide and the HvPap-1 pro-peptide sequence (SPM, PM and P constructs, respectively) in transformed and non-transformed leaves was analyzed by real-time quantitative PCR (RTq-PCR) using specific primers. The level of the mRNAs was normalized to Arabidopsis ubiquitin constitutively expressed transcripts. Strong differences in gene expression among lines were observed (Fig 3A). While high levels of messengers were detected in PM-2.1 and P-3.3 lines, lower mRNA content was found in SPM-1.1, SPM-1.3 and PM-2.2 lines. In general, transgenic leaves expressing the pro-peptide sequence (P construct) showed higher expression levels compared to SPM and PM constructs. No transcripts were found in the RNA isolated from non-transformed Arabidopsis plants.

Transformed and control Arabidopsis lines were also used to analyze the presence of the proteins in leaf extracts by western-blot assays. As shown in Fig 3B, proteins were identified with the anti-GFP antibody and subsequently revealed by a secondary peroxidase conjugated antibody. As expected, proteins of different sizes, 67, 65 and 39 kDa corresponding to the three HvPap-1 protein fragments fused to GFP, were differentially accumulated in lines expressing different transgenes and were absent in the control. Additionally, *in vitro* proteolytic assays were performed with protein extracts derived from Arabidopsis lines. Specific enzymatic activity, expressed as nmol/min/mg, showed lower levels of cathepsin B-like compared to cathepsin L-like activities in transgenic as well as in control plants (Fig 3C). Besides, the cathepsin B-like activity was reduced in most of the transgenic lines compared with the Col control, except in the SPM-line 1.1. Again, variations in the proteolytic patterns were found among different lines, but interestingly, transgenic lines over-expressing the pro-peptide sequence (P lines) presented the strongest decrease in both CysProt activities supporting its potential action as enzyme inhibitor.

### Subcellular localization in Arabidopsis transgenic plants

Transgenic Arabidopsis lines, 14–28 days old, expressing the three independent constructs (SPM, PM and P), containing different fragments derived from the barley *HvPap-1* gene fused to GFP (Fig 2), were analyzed to determine the protein location. Different location patterns were observed depending on the construct used. A diffuse labeling of GFP signal was observed in the membrane system and ER-derived organelles of root cells expressing the entire *HvPap-1* gene-GFP driven by 35S promoter (S3A and S3G Fig). The same construct lacking the signal peptide was clearly and intensively detected at the same location, including nuclear, cytoplasmic and ER membranes of root cells (S3C and S3H Fig). As expected, bright GFP fluorescence was detected at the nuclei and cell cytoplasm of roots transformed with the HvPap-1 pro-peptide-GFP sequence controlled by the 35S promoter (S3E and S3I Fig).

### Spider mite feeding damage on Arabidopsis lines

**Fig 4:**
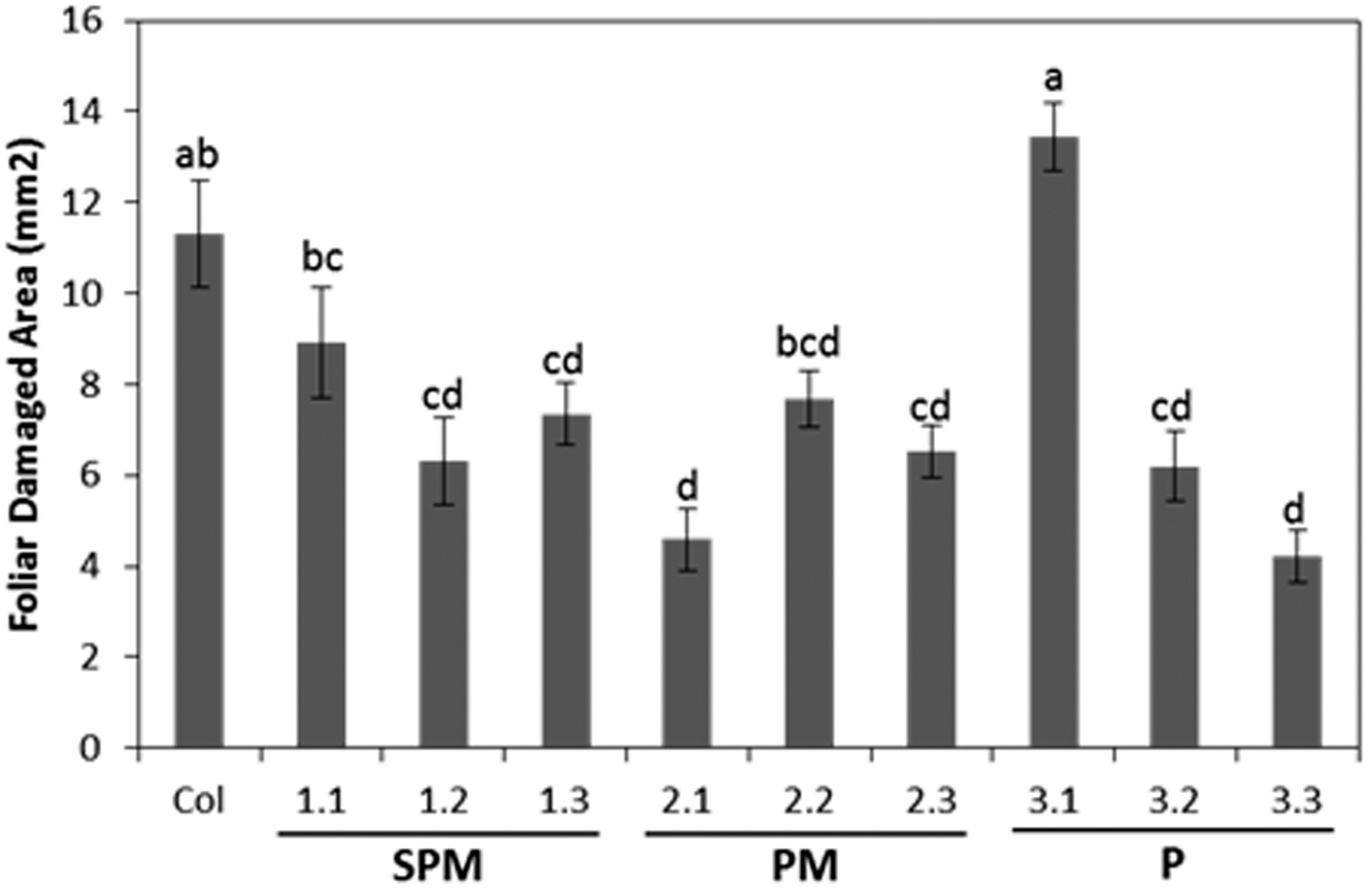
Leaf damage on Arabidopsis transformed and non-transformed lines after *T*. *urticae* infestation. Data are mean ± SE of six measurements. Different letters indicate significant differences (P<0.05, Tukey's HSD). Transgenic plants were: SPM (lines 1.1, 1.2, 1.3), PM (lines 2.1, 2.2, 2.3) and P (lines 3.1, 3.2, 3.3).

To assess the potential defensive effect of the *HvPap-1* gene, *T*. *urticae* was selected to perform bioassays using transformed and non-transformed Arabidopsis lines, containing different fragments derived from the barley *HvPap-1* gene (Fig 2). Leaf damage was quantified after 4 days of mite feeding (Fig 4). The transgenic lines SPM-1.2 and -1.3, PM-2.2 and 2.3 and P-3.2 and 3.3, showed significant less foliar damaged area than control plants. Remarkably, the PM-2.1 and P-3.3 transgenic lines showed 4.5and 4.2 mm^2^ of damaged area, respectively, compared to control plant with 11.3 mm^2^ of average pf damaged area. Exceptionally, a significant injury was observed in the P-3.1 transgenic line produced by mite infestation.

### Effects of Arabidopsis transgenic plants on mites

**Fig 5:**
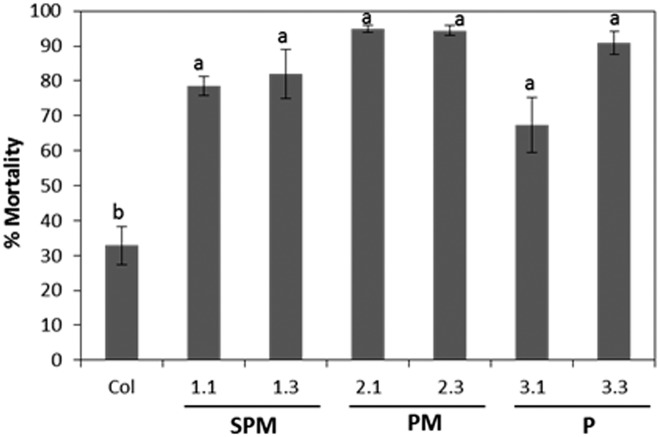
Effects of transgenic Arabidopsis lines expressing different fragments of the *HvPap-1* gene on *T*. *urticae* mortality. Mite mortality expressed as percentage was measured after 10 days of mite of feeding with neonate larvae. Transgenic plants were: SPM (lines 1.1, 1.3), PM (2.1, 2.3) and P (lines 3.1, 3.3). Different letters indicate significant differences (P<0.05, Tukey's HSD test).

T2 transformed and non-transformed Arabidopsis lines were used to investigate the transgene effects on *T*. *urticae*. As shown in Fig 5, mite mortality quantified after 10 days feeding reached values between 67 and 95% when mites fed on transformed lines compared to the 32% on non-transformed plants. Developing time from neonate larvae to nymph lasted 4.88 ± 0.3 days for mites fed on control plants, whereas ranged from 3.97 ± 0.4 to 5.11 ± 0.2 days when fed on transformed lines (S1 Table). These differences observed between transgenic and non-transgenic lines were not statistically significant.

The expression levels of potential mite target transgenes were analysed in adult female mites after 10 days feeding on transformed lines (SPM-3.1, -3.3; PM-2.1, -2.3; and P-3.1, -3.3) and non-transformed Arabidopsis plants (Col). Mite cathepsin L-like (*TuPap49*, *TuPap38*, *TuPap41* and *TuPap42* genes), B-like (*TuPap17* and *TuPap12* genes); D-like (*TuPep2* gene); legumain (*TuLeg5* and *TuLeg10* genes), cystatin (*TuCPI3*, *TuCPI4* genes) and thyropin (*TuThy-1* gene) were selected as the highest expressed genes of each family from RNAseq information available at the ORCAE *T*. *urticae* data base [40]. Among the mite cathepsin L-like genes analysed, induced expression levels were mainly found in mites fed on transgenic plants PM-2.1 and P-3.3 (S4A–S4D Fig). Regarding the two cathepsin B-like genes, both genes were clearly induced on mites fed on PM lines (S4E and S4F Fig). Similarly, the expression of legumain and cathepsin D-like genes was up-regulated in those mites that were fed on the two PM lines (2.1 and 2.3) (S4G–S4I Fig). Curiously, mite genes encoding cystatins, specific inhibitors of CysProt, were also induced in mites after feeding on PM transgenic lines (S4J and S4K Fig) while no differences in thyropin expression were observed in mites fed on different transgenic lines with the exception of the P-3.3 line (S4L Fig).

## Discussion

In previous reports, the *in vitro* inhibitory properties of several recombinant pro-peptides from barley C1A cathepsin L-, B- and F-like against their own enzymes or/and towards commercial proteases were analysed and kinetic assays revealed that all pro-peptides exhibited a competitive inhibition [5, 23]. Now, to obtain further insights on the protective role of these C1A pro-sequences, we selected phytophagous coleopteran and acari species, whose CysProt are the main digestive enzymes in their guts [33, 34, 41–43]. Pro-peptides showed specific inhibitory action against cathepsin B-and L-like activities in all crude extracts from the arthropods tested. However, differences in susceptibility of target enzymes of mite and coleopteran and even among the three mite species and the three coleopteran species were found. Our results suggest that the most appropriated pro-peptide should be specific and dependent on the target arthropod to be controlled. This represents a potential tool and allows the design of accurate strategies using specific rather than general inhibitors to combat specific pests.

Our previous findings identified a proliferation of C1A CysProt in the spider mite *T*. *urticae* genome [38, 39] and demonstrated their susceptibility as targets of cystatins [29, 33]. For this reason, we selected this arthropod to perform bioassays and we chose the *HvPap-1* gene pro-peptide based on the high capability to equally inhibit cathepsin L- and B-like activities in *T*. *urticae* extracts. Transformed plants expressing the entire *HvPap-1* gene, the *HvPap-1* gene without signal peptide and the *HvPap-1* pro-peptide sequence were generated to analyze their putative acaricide effects on *T*. *urticae*. The presence of the transgene transcripts and the corresponding proteins in Arabidopsis lines seemed to be parallel to a reduction in their CysProt proteolytic profiles, particularly in the transgenic lines overexpressing the pro-peptide sequence (P: 3.1, 3.2, 3.3 lines). This data suggested that the HvPap-1 pro-peptide had higher affinity to inhibit C1A cathepsin B-/L-like enzymes when expressed in heterologous systems. Some scenarios can be presented in the *HvPap-1* overexpressing lines: i) inhibition of the expression/activity of other proteases from the same or different class as a result of adapting mechanisms; ii) induction of specific/non-specific protease inhibitors (cystatins) to control protease activity; iii) production of processed pro-peptide with higher affinity to other proteases than its cognate enzyme. This wide range of responses is particularly frequent in protease genes belonging to a multigene family and may produce unexpected results, probably due to their redundant functions.

A key point to compare the pro-peptide effects among transgenic lines is the different location of the specific GFP-constructs used to generate each transgenic line. While SPM and PM lines, containing the *HvPap-1* gene with or without signal peptide, showed similar protein location with differences in the fluorescence intensity, the pro-domain expressed in P lines was located at the nuclei and cell cytoplasm. The protease precursor activation requires appropriated cellular environment for the protease processing [20]. Once the inhibitory pro-domain was claved off from its cognate enzyme, it is ready to specifically bind to other proteases and in consequence inhibit their activities. Thus, differences in specific enzymatic activities found among Arabidopsis lines could be also supported by their different environmental conditions of their sub-cellular location, as was demonstrated in the *ex vivo* processing study performed for maturation of certain Arabidopsis KDEL-CysProt [18].

Feeding trials conducted with the two-spotted spider mite resulted in a significant reduction of leaf damage observed in most of the transgenic lines in comparison to non-transformed control that strongly correlated with an increase in mite mortality. These results demonstrated the susceptibility of mite CysProt to be inhibited by the HvPap-1 pro-peptide and corroborated the crucial role of these enzymes in the mite physiology. The inhibition of the proteolytic processes mediated by this specific pro-peptide may reduce mite access to essential amino acids. Consequently, protein function can be impaired disrupting mite physiological events and, in consequence, increasing mortality. Similar results were shown after performing mite feeding bioassays on double transformed Arabidopsis lines expressing simultaneously cysteine- and serine-protease inhibitors (barley cystatin 6 plus CMe inhibitor). In this case, a parallel retardation tendency in the larvae development corroborated the role of the serine proteases in the spider mite growth [29]. In contrast, no effects of transgenic plants were observed on mite developmental process determined as time needed by *T*. *urticae* larvae to reach the adult stage *in planta*. Probably, the HvPap-1 pro-peptide preferentially inhibited CysProt involved in mite digestion. Additionally, our data are in agreement with the results described by Visal et al. [24] which demonstrated that the pro-region of papaya proteinase IV inhibited digestive CysProt of the Colorado potato beetle (*L*. *decemlineata*), and particularly with the protection against nematode infestation conferred by the transgenic expression of the CysProt pro-region from *H*. *glycines* [27]. Furthermore, not only phytophagous mites or insects can be controlled by inhibitory peptides, in previous works have been shown that pro-domains of other protease families from different origin efficiently inhibited enzymes of parasites and pathogens [26, 44, 45]. In this scenario, we decided to analyze mite responses after feeding on transgenic lines by analyzing the expression levels of a selection of potential mite targets. Curiously, we found that mites fed on PM-2.1 and P-3.3. lines (that presented less foliar damage but produced the highest mite mortality) showed a high induction of the four selected cathepsin L-like genes. Additionally, mite genes encoding cathepsin B-like, legumain and cystatin were also up-regulated after feeding on PM-2.1, 2.3 and P-3.3 lines. The over-expression of targeted proteases, the induction of other non-targeted enzymes or even the activation of protease inhibitors have been described as a common strategy to counteract inhibitory activities [29, 46, 47].

It is well documented that C1A CysProt have performed crucial defence roles in pathogen/pest interactions through different mechanisms of action [10–13, 42]. The potential interest of the pro-peptides as part of the CysProt has appeared as an alternative to control pest in those cases where Bt genes resulted unsuccessful such as against mite pests [37, 48]. Furthermore, pro-peptide sequence can act as defense transgenes when integrated into gene pyramiding strategies. They could exert similar mechanism of action than cystatins or other related molecules [29, 33, 49, 50].

## Conclusions

- C1A CysProt pro-peptides from barley, expressed as recombinant protein, specifically inhibited cathepsin L- and B-like proteolytic activities of phytophagous coleopteran (*L*. *decemlineata*, *D*. *virgifera* and *T*. *molitor*) and phytophagous acari (*T*. *urticae*, *T*. *evansi* and *B*. *chilensis*).
- The stable expression of the CysProt HvPap-1 pro-peptide from barley in Arabidopsis confers protection against *T*. *urticae* spider mite feeding.
- *T*. *urticae* spider mites try to counteract the HvPap-1 pro-peptide inhibitory action by over-expressing CysProt of different classes, including C1A and C13 families.
- CysProt pro-peptides seem to be promising proteins to control mites and insect pests in biotechnological systems, becoming in a potential tool to design accurate strategies dependent on the target arthropod to be controlled.

## Materials and Methods

### Expression and purification of recombinant pro-peptides from *E*. *coli*

cDNA fragments spanning the putative pro-peptide regions *HvPap-1*, *-4*, *-6*, *-10*, *-12*, *16*, *-17* and *-19* genes [2] were amplified and cloned into expression vector pRSETB as described [5, 23]. The corresponding recombinant CysProt pro-peptides (HvPap-1pro, HvPap-4pro, HvPap-6pro, HvPap-10pro, HvPap-12pro, HvPap-16pro, HvPap-17pro and HvPap-19pro) were expressed and purified according to Martinez et al. [3]. Alignment of the amino acid pro-peptide sequences was performed using the default parameters of MUSCLE (S1 Fig).

### Inhibitory activity of pro-peptides against phytophagous arthropods

Three coleopteran (*L*. *decemlineata*, *T*. *molitor* and *D*. *virgifera*) and three acari (*T*. *urticae*, *T*. *evansi* and *B*. *chilensis)* species were selected to analyze pro-peptide inhibitory activities. Protein extracts from *T*. *molitor* larvae, *L*. *decemlineata* isolated guts, lyophilized samples of *D*. *virgifera* (gifts of Dr. Ortego, CIB-CSIC, Spain); and lyophilized samples of *B*. *chilensis* (gift of Dr. Gambardella, PUC, Chile) were isolated. Colonies of *T*. *urticae* (London strain) and *T*. *evansi* (Beausoleil strain) reared on *Phaseolus vulgaris* and *Solanum nigrum* plants, respectively, in our laboratory at 25°C, 70% relative humidity and 16h/8h day/night photoperiod, were also used to extract total proteins. All arthropod samples were homogenized in 0.15 M NaCl, centrifuged at 10,000 rpm for 5 min and the supernatants were used to quantify protein content using a Nanodrop ND. The inhibitory activity of the eight recombinant pro-peptides was *in vitro* tested against the different arthropod proteins. The standard assay volume was 100 μL, containing 2 μg of arthropod proteins in a buffer A (100 mM sodium phosphate pH 6.0, L-cysteine, 10 mM EDTA and 0.01% Brij35). Different amounts of recombinant pro-peptides (1.0 μg of HvPap-1pro and HvPap-16pro; 0.5 μg of HvPap-4pro, HvPap-10pro, HvPap-12pro, HvPap-17pro and HvPap-19pro; and 0.1 μg of HvPap-6pro) were used, based on the *Ki* values previously obtained against commercial cathepsins [5, 23]. After a pre-incubation at 28°C for 20 min, the substrates Z-FR-AMC (N-carbobenzoxyloxy-Phe-Arg-7-amido-4-methyl coumarin) and Z-RR-AMC (N-carbobenzoxyloxy-Arg-Arg-7-amido-4-methyl coumarin) were added to a final concentration of 25 μM to assess the inhibition of cathepsin L-like and B-like activities, respectively. Reactions were incubated for 2.5 h at 28°C and fluorescence was measured with a 365 nm excitation filter and 465 nm emission filter. Assays were performed in triplicate and blanks were used to account for the spontaneous breakdown of substrates. Results are expressed as percentages of inhibition of cathepsin L- and B-like activities.

### Plasmid constructs and plant transformation

Three different fragments spanning the whole *HvPap-1* ORF including the signal peptide sequenced from barley (*Hordeum vulgare*) were amplified using specific primers (S2 Table). The three constructs contained: i) the entire *HvPap-1* gene containing the signal peptide, the N-terminal pro-peptide and the mature protein (construct SPM); ii) the *HvPap-1* gene without signal peptide (construct PM); and iii) the *HvPap-1* pro-peptide sequence (construct P). The *HvPap-1* ORF derived fragments were cloned into a Gateway binary vector pGWB5 (Invitrogen) under the CaMV35S promoter and fused to the *GFP* gene (Fig 2). Transgenic *A*. *thaliana* Columbia (Col) were generated by the *Agrobacterium*-mediated floral dip method [51]. Transgenic and non-transformed Col plants were grown under controlled conditions at 22°C, 70% relative humidity and 16h/8h day/night photoperiod. Arabidopsis seeds from transgenic plants were harvested and plated onto 1/2 MS medium containing 50 μg/ml hygromycin and the resultant seedlings were transplanted to soil and allowed to set seeds. The genotype of the transgenic plants was determined by a segregation test using the seed progenies and growing the T2 transgenic plants on a 1/2 MS solid medium with hygromycin (50 μg/ml). T2 homozygous seeds were selected for further characterization and mite bioassays.

### Nucleic acid analysis

Total DNA was isolated from control and T2 selected transgenic lines (SPM: 1.1, 1.2 and 1.3; PM: 2.1, 2.2 and 2.3; P: 3.1, 3.2 and 3.3). The presence of the transgene sequences was analyzed by conventional PCR using specific primers (S2 Table) and particular reaction conditions (40 cycles with 30 sec at 95°C, 80 sec, 70 sec or 40 sec at 60°C for SPM, PM and P constructs, respectively, plus 80 sec at 72°C). Amplified products were separated on 1% agarose electrophoresis gels. For RTq-PCR assays, total RNA was extracted from control and transgenic leaves by the phenol/chloroform method [52]. RNA was also prepared from *T*. *urticae* after 2 days of feeding on leaf disks from control and transgenic Arabidopsis lines. Total RNA was purified with TRiZOL (Ambion) following manufacturer instructions, and some modifications previously described by Santamaria et al. [29]. cDNAs were synthesized from 2 μg of RNA using the Revert Aid H Minus First Strand cDNA Synthesis Kit (Thermo Scientific Fermentas) following manufacturer instructions. RTq-PCR conditions were 45 cycles with 15 sec at 95°C, 1 min at 55°C and 5 sec at 65°C. FastStart Universal SYBR Green Master (Roche) was used in a total volume of 20 μl. For negative controls, H_2_O was used instead of cDNA. PCR reactions, performed in multiplate PCR plates (BioRad), were carried out in a C1000 thermal cycler with CFX96 optical reaction module (BioRad) and results were analyzed using CFX Manager Software 2.0 (BioRad). Triplicate assays were done and quantification was standardized to ubiquitin or Rp49 mRNA levels for plant and spider mite samples, respectively. Gene expression values were referred as relative expression (2^-dCt^) and fold change (2^-ddCt^) for plant and *T*. *urticae* samples respectively. Primers used to perform Arabidopsis and *T*. *urticae* RT-qPCR are in S3 and S4 Tables, respectively.

### Protein detection

Leaves from transgenic and control Arabidopsis plants were ground and resuspended in extraction buffer (0.15 M NaCl, 50 mM sodium phosphate pH 6.0, 2 mM EDTA). After centrifugation at 10,000 rpm for 5 min, supernatants were recovered and stored at -20°C. Total protein concentration was determined by the method of Bradford [53] using the Bio-Rad protein assay (Bio-Rad Laboratories, Germany) with Bovine Serum Albumin (BSA) as standard. 10 μg of protein extracts were separated on SDS-plyacrylamide gels (12–15% w/v) according to Laemmli (1970) [54] and electro-transferred onto nitrocellulose membrane (Amersham Protean, GE Healthcare). Membrane was blocked with 3% BSA in phosphate-buffered saline with 0.05% Tween (PBST) and incubated with the commercial anti-GFP polyclonal antibody (1:1000, v:v) produced by Roche. Alternatively, it was used the commercial antibody against to the large subunit of Rubisco (1:1000, v:v) produced by Agrisera. After several washes, membrane was probed with peroxidase conjugated anti-rabbit IgG (1:10000, v:v), for detection with SuperSignal Detection Kit (Pierce).

### Enzymatic activities

Enzymatic activities of plant protein extracts were *in vitro* tested using Z-FR-AMC and Z-RR-AMC as substrates of cathepsin L- and B-like proteases, respectively. Assays were carried out in microplates. 2 μg of protein extracts were incubated with the substrate at a final concentration of 25 μM using the buffer A. Hydrolysis of substrates containing the AMC (7-amido-4-methyl coumarin) fluorophore was measured to detect cathepsin B- and L-like activities. Specific enzymatic activity was calculated as nmoles of substrate hydrolyzed/min/mg protein. All assays were carried out in triplicate and blanks were used to account for spontaneous breakdown of substrates.

### Subcellular location of *HvPap-1* gene fragments

Homozygous Arabidopsis lines expressing SPM, PM or P constructs, described above, were selected to analyse transgene location. Hypocotyl, leaf and root samples were observed and finally, fluorescence images from roots were taken using a fluorescence microscope Zeiss Axiophot.

### Leaf damage quantification on Arabidopsis plants after mite feeding

Damage quantification was done on Arabidopsis control and T2 transgenic lines (SPM: lines 1.1, 1.2 and 1.3; PM: lines 2.1, 2.2 and 2.3 and P: lines 3.1, 3.2 and 3.3) after spider mite feeding. Three week old plants were infested with 20 adults of *T*. *urticae* per plant. 4 days after infestation, the leaf damage was assessed according to Santamaria et al. [29]. Damaged leaves were scanned using a scanner (HP Scanjet 5590 Digital Flatbed Scanner) and injury was calculated as mm^2^ of affected tissue using Adobe Photoshop CS software according to Cazaux et al. [55]. Six replicates per plant per transgenic line and non-transformed control were done.

### Mite bioassays on Arabidopsis plants

Entire detached leaves from control and selected T2 transgenic lines (SPM: 1.1 and 1.3; PM: 2.1 and 2.3; P: 3.1 and 3.3) were used to carry out spider mite bioassays. A special confined structure was designed to perform the mite developmental and mortality tests. Briefly, a small Petri dish (Ø = 4 cm) with a ventilation system was separated in two parts by a parafilm layer. The down part of the plate was filled with water through a hole in the parafilm layer. Entire leaf was placed across the parafilm, remaining the petiole in touch with the water. Eggs from *T*. *urticae* were collected using sieves according to Cazaux et al. [55]. Approximately, one hundred eggs were placed on the detached leaf. To avoid mite escapes, dishes were sealed with a parafilm layer around the edge. Samples were maintained under controlled conditions at 25°C, 70% relative humidity and 16h/8h day/night photoperiod. Next day, neonate larvae were counted to start the development experiment. During the following 10 days, larvae become neo-nymph or death larvae were counted day by day, in order to calculate both developmental and mortality rates. New fresh leaf from new plants was added every 2 days of assay. Results are represented as percentages of mortality and days needed for larvae to become neo-nymph. Six replicates per transgenic line and non-transformed control were done.

### Statistical analysis

Differences in inhibitory and enzymatic activities, leaf damage, mite mortality and gene expression levels were compared by one-way ANOVA, followed by studentized range distribution of Tukey's HSD (honest significant difference) test using R statistic software.

## Supporting Information

S1 Fig Alignment of the amino acid pro-peptide sequences. Putative pro-peptides of HvPap-1, -4, -6, -10, -12, -16, -17 and -19 proteins were aligned by MUSCLE program.
(PPTX)

S2 Fig PCR analysis of T2 transgenic and non-transformed Arabidopsis lines. Genomic PCR was performed using specific primers described in the Table S2. Transgenic plants were: SPM (lines 1.1, 1.2, 1.3); PM (lines 2.1, 2.2, 2.3); P (lines 3.1, 3.2, 3.3) and non-transformed control (Col). M: 100 bp molecular size marker. Numbers indicate the size of the corresponding amplified fragments.
(PPTX)

S3 Fig Location of *HvPap-1* gene fragments fused to GFP in transformed Arabidopsis lines. Location of the entire *HvPap-1* gene (A and G), the *HvPap-1* gene lacking the signal peptide (C and H) and the *HvPap-1* pro-peptide sequence (E and I) in mature root cells. Same images were taken under light field conditions (B, D, F). Transgenic plants were: SPM, PM and P lines. Similar images were acquired from three independent transgenic plants. Scale bars: 75 μm (A-I).
(PPTX)

S4 Fig Mite gene expression levels after feeding on transformed and control Arabidopsis lines. Adult female mite genes encoding cathepsin L-like (A,B,C,D); cathepsin B-like (E,F); legumain (G,H); cathepsin D-like (I); cystatin (J, K) and thyropin (L) were analysed after 10 days feeding on transformed and non-transformed Arabidopsis lines. Transgenic plants were: SPM plants (lines 1.1, 1.3), PM plants (lines 2.1, 2.3), P plants (lines 3.1, 3.3) and non-transformed control (Col). Data were the mean ± SE of two replicates for each sample. Different letters indicate significant differences (P<0.05, HSD test).
(PPTX)

S1 Table Effects of the transgenic Arabidopsis lines on *T*. *urticae* development after feeding bioassay. (DOCX)

S2 Table Oligonucleotide primers used for conventional PCR of barley *HvPap-1* gene. (DOCX)

S3 Table Oligonucleotide primers used for RTq-PCR of *Arabidopsis* transgenic lines. (DOCX)

S4 Table Oligonucleotide primers used for RTq-PCR of *T*. *urticae* genes. (DOCX)

## References

[1] Rawlings ND, Waller M, Barrett AJ, Bateman A (2014) MEROPS: the database of proteolytic enzymes, their substrates and inhibitors. Nucleic Acids Res 42: D503–D509. 10.1093/nar/gkt953 24157837 PMC3964991

[2] Martinez M, Diaz I (2008) The origin and evolution of plant cystatins and their target cysteine proteinases indicate a complex functional relationship. BMC Evol Biol 8: 198 10.1186/1471-2148-8-198 18616807 PMC2474614

[3] Martinez M, Cambra I, Carrillo L, Diaz-Mendoza M, Diaz I (2009) Characterization of the entire cystatin gene family in barley and their target cathepsin L-Like cysteine-proteases, partners in the hordein mobilization during seed germination. Plant Physiol 151: 1531–1545. 10.1104/pp.109.146019 19759340 PMC2773090

[4] Parrott DL, Martin JM, Fischer AM (2010) Analysis of barley (*Hordeum vulgare*) leaf senescence and protease gene expression: a family C1A cysteine protease is specifically induced under conditions characterized by high carbohydrate, but low to moderate nitrogen levels. New Phytol 187: 313–31. 10.1111/j.1469-8137.2010.03278.x 20456047

[5] Cambra I, Martinez M, Dader B, González-Melendi P, Gandullo J, Santamaría ME, et al (2012b) A cathepsin F-like peptidase involved in barley grain protein mobilization, HvPap-1, is modulated by its own propeptide and by cystatins. J Exp Bot 63: 4615–4629. 10.1093/jxb/ers137 22791822 PMC3421991

[6] Diaz-Mendoza M, Arroyo-Velasco B, Gonzalez-Melendi P, Martinez M, Diaz I (2014) C1A cysteine protease-cystatin interactions in leaf senescence. J Exp Bot 65: 3825–3833. 10.1093/jxb/eru043 24600023

[7] Grudkowska M, Zagdanska B (2004) Multifunctional role of plant cysteine proteinases. Acta Biochim Pol 51: 609–624. 15448724

[8] Diaz I, Martinez M (2013) Plant C1A cysteine peptidases in germination and senescence In: Rawlings ND, Salvesen GS, editors. Handbook of proteolytic enzymes. Amsterdam: Elsevier pp. 1852–1858.

[9] Zhang D, Liu D, Lu X, Wang Y, Xun Z, Liu Z, et al (2014) The cysteine protease CEP1, a key executor involved in tapetal programmed cell death, regulates pollen development in Arabidopsis. Plant Cell 26: 2939–2961. 10.1105/tpc.114.127282 25035401 PMC4145124

[10] McLellan H, Gilroy EM, Yun BW, Birch PRJ, Loake GJ (2009) Functional redundancy in the Arabidopsis cathepsin B gene family contributes to basal defence, the hypersensitive response and senescence. New Phytol 183: 408–418. 10.1111/j.1469-8137.2009.02865.x 19453434

[11] Harrison RL, Bonning BC (2010) Proteases as insecticidal agents Toxins 2: 935–953. 10.3390/toxins2050935 22069618 PMC3153225

[12] Martinez M, Cambra I, Gonzalez-Melendi P, Santamaria ME, Diaz I (2012) C1A cysteine-proteases and their inhibitors in plants. Physiol Plant 145: 85–94. 10.1111/j.1399-3054.2012.01569.x 22221156

[13] Wang W, Zhang L, Guo N, Zhang X, Zhang C, Sun G, et al (2014) Functional properties of a cysteine proteinase from pineapple fruit with improved resistance to fungal pathogens in *Arabidopsis thaliana* . Molecules 19: 2374–2389. 10.3390/molecules19022374 24566309 PMC6271751

[14] Okamoto T, Shimada T, Hara-Nishimura I, Nishimura M, Minamikawa T (2003) C-terminal KDEL sequence of a KDEL-tailed cysteine-proteinase (sulfhydryl-endopeptidase) is involved in formation of KDEL vesicle in efficient vacuolar transport of sulfhydryl-endopeptidase. Plant Physiol 132: 1892–1900. 12913146 10.1104/pp.103.021147 PMC181275

[15] Helm M, Hierl G, Terneus K, Tan L, Lottspeich F, Kieliszewski MJ, et al (2008) KDEL-tailed cysteine endopeptidases involved in programmed cell death, intercalation of new cells, and dismantling of extension scaffolds. Am J Bot 95: 1049–1062. 10.3732/ajb.2007404 21632425

[16] Carrion CA, Costa ML, Martinez DE, Mohr C, Humbeck K, Guiamet JJ (2013) *In vivo* inhibition of cysteine proteases provides evidence for the involvement of “senescence-associated vacuoles” in chloroplast protein degradation during dark-induced senescence of tobacco leaves. J Exp Bot 64: 4967–4980. 10.1093/jxb/ert285 24106291

[17] Schmidt M, Simpson DJ, Srioglu H, Lottspeich F, Gietl C (2001) The ricinosomes of senescing plant tissue bud from the endoplasmic reticulum. Proc Natl Acad Sci USA 98: 5353–5358. 11296243 10.1073/pnas.061038298 PMC33213

[18] Hierl G, Howing T, Isono E, Lottspeich F, Gietl C (2014) *Ex vivo* processing for maturation of Arabidopsis KDEL-tailed cysteine endopeptidase 2 (AtCEP2) pro-enzyme and its storage in endoplasmic reticulum derived organelles. Plant Mol Biol 84: 605–620. 10.1007/s11103-013-0157-6 24287716 PMC3950626

[19] Wiederanders B (2003) Structure-function relationship in class C1A cysteine peptidases. Acta Biochim Pol 50: 691–713. 14515150

[20] Demidyuk IV, Shubin AV, Casanov EV, Kostrov SV (2010) Propeptides as modulators of functional activity of proteases. BioMol Concepts 1: 305–322. 10.1515/bmc.2010.025 25962005

[21] Taylor MA, Baker KC, Briggs CS, Connerton IF, Cummings NJ, Pratt KA, et al (1995) Recombinant pro-region from papain and papaya proteinase IV-are selective high affinity inhibitors of the mature papaya enzymes. Protein Eng 8: 59–62. 7770454 10.1093/protein/8.1.59

[22] Gutierrez-Gonzalez LH, Rojo-Dominguez A, Cabrera-Gonzalez NE, Perez-Monfort R, Padilla-Zuniga AJ (2006) Losely packed papain prosegment displays inhibitory activity. Arch Biochem Biophys 446: 151–160. 16427023 10.1016/j.abb.2005.12.005

[23] Cambra I, Hernandez D, Diaz I, Martinez M (2012a) Structural basis for specificity of propeptide-enzyme interaction in barley C1A cysteine peptidases. PLoS One 7: e37234 10.1371/journal.pone.0037234 22615948 PMC3355106

[24] Visal S, Taylor MAJ, Michaud D (1998) The proregion of papaya proteinase IV inhibits Colorado potato beetle digestive cysteine proteinases. FEBS Lett 434: 401–405. 9742962 10.1016/s0014-5793(98)01018-7

[25] Silva FB, Batista JAN, Marra BM, Fragoso RR, Monteiro ACS, Figueira ELD, et al (2004) Prodomain peptide of HGCP-Iv cysteine proteinase inhibits nematode cysteine proteinases. Genet Mol Res 3: 342–355. 15614726

[26] Silva ACS, Monteiro RP, Del Sarto BM, Marra SC, Dias ELZ, Figueira GR, et al (2007) Proregion of *Acanthoscelides obtectus* cysteine proteinase: A novel peptide with enhanced selectivity toward endogenous enzymes. Peptides 28: 1292–1298. 17485144 10.1016/j.peptides.2007.03.020

[27] Marra BM, Souza DSL, Aguiar JN, Firmino AAP, Sarto RPD, Silva FB, et al (2009) Protective effects of a cysteine proteinase propeptide expressed in transgenic soybean roots. Peptides 30: 825–831. 10.1016/j.peptides.2009.01.022 19428757

[28] Benchabane M, Schlüter U, Vorster J, Goulet MC, Michaud D (2010) Plant cystatins. Biochimie 92: 1657–1666. 10.1016/j.biochi.2010.06.006 20558232

[29] Santamaria ME, Cambra I, Martinez M, Pozancos C, Gonzalez-Melendi P, Grbic V, et al (2012a) Gene pyramiding of peptidase inhibitors enhances plant resistance to the spider mite *Tetranychus urticae* . PLoS One 7: e43011 10.1371/journal.pone.0043011 22900081 PMC3416837

[30] Lima AM, Dos Reis SP, de Souza CR (2014) Phytocystatins and their potential to control plant diseases caused by fungi. Protein Pep Lett (in press) 10.2174/0929866521666140418101711 24746092

[31] Ortego F (2012) Physiology adaptations of the insect gut to herbivory In: Smagghe G, Diaz I, editors. Arthropod-plant interactions. Springer, pp.75–88.

[32] Novillo C, Castañera P, Ortego F (1997) Characterization and distribution of chymotrypsin-like and other digestive proteases in Colorado potato beetle larvae. Arch Insect Biochem Physiol 36: 181–201.

[33] Carrillo L, Martinez M, Ramessar K, Cambra I, Castañera P, Ortego F, et al (2011) Expression of a barley cystatin gene in maize enhances resistance against phytophagous mites by altering their cysteine-proteases. Plant Cell Rep 30: 101–112. 10.1007/s00299-010-0948-z 21082183

[34] Kim JH, Mullin CA (2003) Impact of cysteine proteinase inhibition in midgut fluid and oral secretion on fecundity and pollen consumption of western corn rootworm (*Diabrotica virgifera virgifera*). Arch Insect Biochem Phys 52: 139–154. 12587142 10.1002/arch.10074

[35] Migeon A, Dorkeld F (2011) Spider Mites Web: a comprehensive database for the Tetranychidae. Available: http://www.montpellier.inra.fr/CBGP/spmweb.

[36] Van Leeuwen T, Demaeght P, Osborne EJ, Dermauw W, Gohlke S, Nauen R, et al (2012) Population bulk segregant mapping uncovers resistance mutations and the mode of action of a chitin synthesis inhibitor in arthropods. Proc Natl Acad Sci USA 105: 598–5985. 10.1073/pnas.1200068109 PMC3311382 22393009

[37] Rovenska GZ, Zemek R, Schmidt JEU, Hilbeck A (2005) Altered host plant preference of *Tetranychus urticae* and prey preference of its predator *Phytoseiulus persimilis* (Acari: Tetranychidae, Phytoseiidae) on transgenic Cry3Bb-eggplants. Biol Control 33: 293–300. 15781137

[38] Grbic M, Van Leeuwen T, Clark R, Rombauts S, Rouze P, Grbic V, et al (2011) The genome of *Tetranychus urticae* reveals herbivorous pest adaptations. Nature 479: 487–492. 10.1038/nature10640 22113690 PMC4856440

[39] Santamaria ME, Hernández-Crespo P, Ortego F, Grbic V, Grbic M, Diaz I, et al (2012b) Cysteine peptidases and their inhibitors in *Tetranychus urticae*: a comparative genomic approach. BMC Genom 13: 307. 10.1186/1471-2164-13-307 PMC3407033 22784002

[40] ORCAE (Online Resource for Community Annotation of Eukaryotes) Tetranychus urticae website Available: http://bioinformatics.psb.ugent.be/orcae/overview/Tetur). 10.1038/nmeth.2242 23132114

[41] Thie NMR, Houseman JG (1997) identification of cathepsin B, D, and H in the larval midgut of Colorado potato beetle *Leptinotarsa decemlineata* Say (Coleoptera: Chrysomelidae). Insect Biochem 20: 313–318.

[42] Shindo T, van der Hoorn RA (2008) Papain-like cysteine proteases: key players at molecular battlefields employed by both plants and their invaders. Mol Plant Pathol 9: 119–125. 10.1111/j.1364-3703.2007.00439.x 18705889 PMC6640327

[43] Vinokurov SK, Elpidina EN, Oppert B, Prabhakar S, Zhuzhikov DP, Dunaevsky YE, et al (2006) Fractionation of digestive proteinases from Tenebrio molitor (Coleoptera: Tenebrionidae) larvae and role in protein digestion. Comp Biochem Phys Part B: Biochem Mol Biol 145: 138–146. 16926103 10.1016/j.cbpb.2006.05.004

[44] Taylor MA, Lee MJ (1997) Trypsin isolated from the midgut of the tobacco hornworn, *Manduca sexta* is inhibited by synthetic pro-peptides in vitro Biochem Biophys Res Commun 135: 606–609. 10.1006/bbrc.1997.6839 9207205

[45] Pandey KC, Barkan DT, Sali A, Rosenthal PJ (2009) Regulatory elements within the prodomain of Falcipain-2, a cysteine protease of the malaria parasite *Plasmodium falciparum* . PLoS One 4:e4694 10.1371/journal.pone.0004694 19479029 PMC2682653

[46] Lara P, Ortego F, Gonzalez-Hidalgo E, Castañera P, Carbonero P, Diaz I (2000) Adaptation of *Spodoptera exigua* (Lepidoptera: Noctuidiae) to barley trypsin inhibitor BTI-CMe expressed in transgenic tobacco. Transgenic Res 9: 169–178. 11032365 10.1023/a:1008905631440

[47] Alvarez-Alfageme F, Martinez M, Pascual-Ruiz S, Castañera P, Diaz I, Ortego F (2007) Effects of potato plants expressing a barley cystatin on the predatory bug *Podisus maculiventris* via herbivorous prey feeding on the plant. Transgenic Res 16: 1–13. 17072562 10.1007/s11248-006-9022-6

[48] Li Y, Romeis J (2010) Bt maize expressing Cry3Bb1 does not harm the spider mite, *Tetranychus urticae*, or its ladybird beetle predator, *Stethorus punctillum* . Biol Control 56: 157–164.

[49] McCafferty HRK, Moore PH, Zhu Y (2006) Improved *Carica papaya* tolerance to carmine spider mite by the expression of *Manduca sexta* chitinase transgene. Transgenic Res 15: 337–347. 16779649 10.1007/s11248-006-0005-4

[50] McCafferty HRK, Moore PH, Zhu Y (2008) Papaya transformed with the *Galanthus nivalis* GNA gene produces a biologically active lectin with spider mite control activity. Plant Sci 175: 385–393.

[51] Clough SJ, Bent AF (1998) Floral dip: a simplified method for *Agrobacterium*-mediated transformation of *Arabidopsis thaliana* . Plant J 16: 735–743. 10069079 10.1046/j.1365-313x.1998.00343.x

[52] Oñate-Sanchez L, Vicente-Carbajosa J (2008) DNA-free RNA isolation protocols for *Arabidopsis thaliana*, including seeds and siliques. BMC Res Notes 1: 93 10.1186/1756-0500-1-93 18937828 PMC2613888

[53] Bradford MM (1976) A rapid and sensitive method for the quantification of microgram quantities of protein utilizing the principle of protein-dye binding. Ann Biochem 72: 248–254. 10.1016/0003-2697(76)90527-3 942051

[54] Laemmli UK (1970) Cleavage of structural proteins during assembly of head of bacteriophage T4. Nature 277: 680–685. 10.1038/227680a0 5432063

[55] Cazaux M, Navarro M, Bruinsma KA, Zhurov V, Negrave T, Van Leeuwen T, et al (2014) Application of two-spotted spider mite *Tetranychus urticae* for plant-pest interaction studies. J Vis Exp 89: e51738. 10.3791/51738 PMC4211727 25046103

