# Supplementary material for: Inhibitory Properties of Cysteine Protease Pro-Peptides from Barley Confer Resistance to Spider Mite Feeding
Source: PLoS One. 2015 Jun 3;10(6):e0128323. doi: 10.1371/journal.pone.0128323 (PMC4454591; doi:10.1371/journal.pone.0128323)
Supplement: S1 Fig — Putative pro-peptides of HvPap-1, -4, -6, -10, -12, -16, -17 and -19 proteins were aligned by MUSCLE program. (PPTX) [file pone.0128323.s001.pptx]

## Slide 1
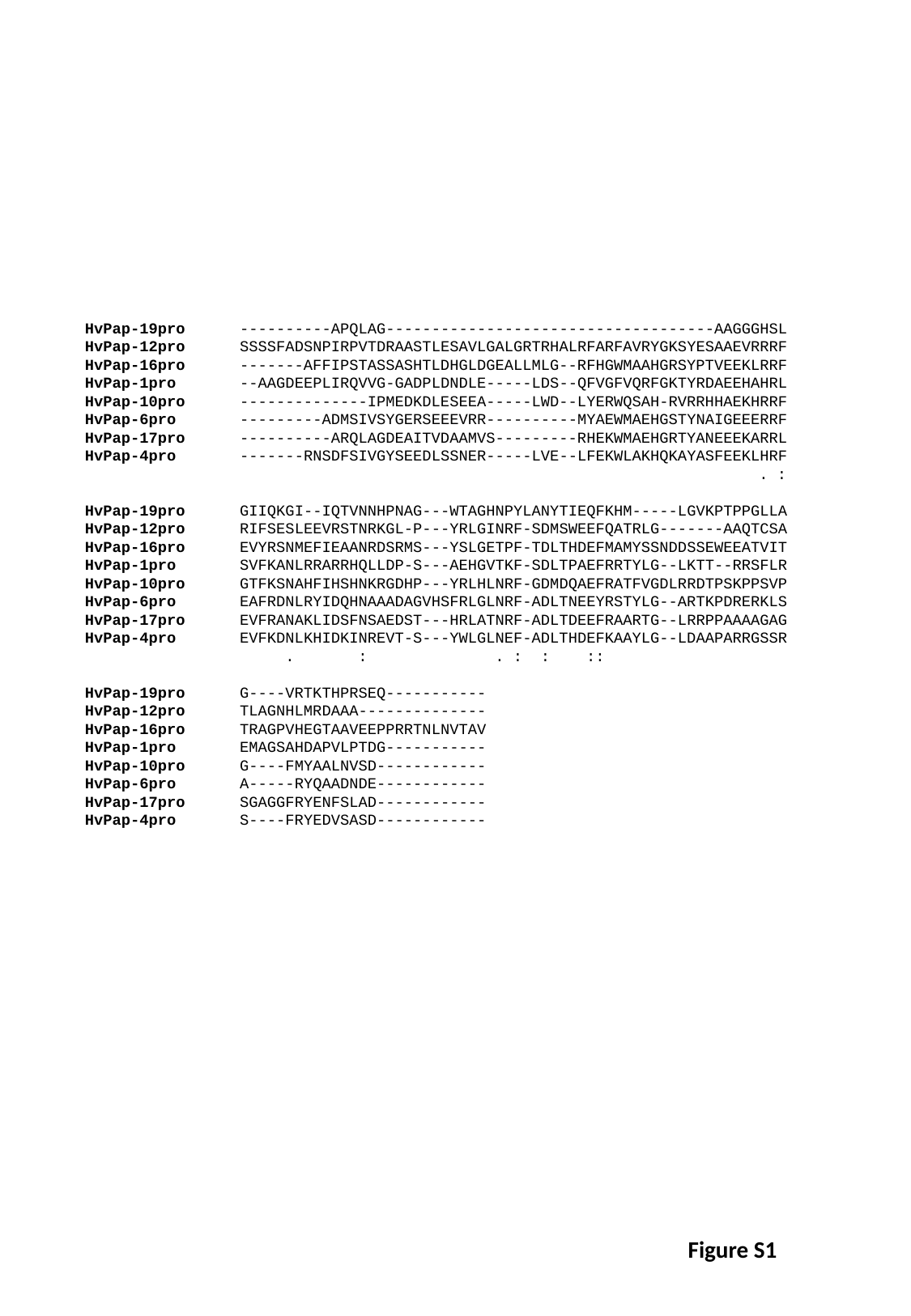

HvPap-19pro ----------APQLAG------------------------------------AAGGGHSL
HvPap-12pro SSSSFADSNPIRPVTDRAASTLESAVLGALGRTRHALRFARFAVRYGKSYESAAEVRRRF
HvPap-16pro -------AFFIPSTASSASHTLDHGLDGEALLMLG--RFHGWMAAHGRSYPTVEEKLRRF
HvPap-1pro --AAGDEEPLIRQVVG-GADPLDNDLE-----LDS--QFVGFVQRFGKTYRDAEEHAHRL
HvPap-10pro --------------IPMEDKDLESEEA-----LWD--LYERWQSAH-RVRRHHAEKHRRF
HvPap-6pro ---------ADMSIVSYGERSEEEVRR----------MYAEWMAEHGSTYNAIGEEERRF
HvPap-17pro ----------ARQLAGDEAITVDAAMVS---------RHEKWMAEHGRTYANEEEKARRL
HvPap-4pro -------RNSDFSIVGYSEEDLSSNER-----LVE--LFEKWLAKHQKAYASFEEKLHRF
 . :
HvPap-19pro GIIQKGI--IQTVNNHPNAG---WTAGHNPYLANYTIEQFKHM-----LGVKPTPPGLLA
HvPap-12pro RIFSESLEEVRSTNRKGL-P---YRLGINRF-SDMSWEEFQATRLG-------AAQTCSA
HvPap-16pro EVYRSNMEFIEAANRDSRMS---YSLGETPF-TDLTHDEFMAMYSSNDDSSEWEEATVIT
HvPap-1pro SVFKANLRRARRHQLLDP-S---AEHGVTKF-SDLTPAEFRRTYLG--LKTT--RRSFLR
HvPap-10pro GTFKSNAHFIHSHNKRGDHP---YRLHLNRF-GDMDQAEFRATFVGDLRRDTPSKPPSVP
HvPap-6pro EAFRDNLRYIDQHNAAADAGVHSFRLGLNRF-ADLTNEEYRSTYLG--ARTKPDRERKLS
HvPap-17pro EVFRANAKLIDSFNSAEDST---HRLATNRF-ADLTDEEFRAARTG--LRRPPAAAAGAG
HvPap-4pro EVFKDNLKHIDKINREVT-S---YWLGLNEF-ADLTHDEFKAAYLG--LDAAPARRGSSR
 . : . : : ::
HvPap-19pro G----VRTKTHPRSEQ-----------
HvPap-12pro TLAGNHLMRDAAA--------------
HvPap-16pro TRAGPVHEGTAAVEEPPRRTNLNVTAV
HvPap-1pro EMAGSAHDAPVLPTDG-----------
HvPap-10pro G----FMYAALNVSD------------
HvPap-6pro A-----RYQAADNDE------------
HvPap-17pro SGAGGFRYENFSLAD------------
HvPap-4pro S----FRYEDVSASD------------
Figure S1
