# Supplementary figures and images for: Inhibitory Properties of Cysteine Protease Pro-Peptides from Barley Confer Resistance to Spider Mite Feeding
Source: PLoS One. 2015 Jun 3;10(6):e0128323. doi: 10.1371/journal.pone.0128323 (PMC4454591; doi:10.1371/journal.pone.0128323)

## Slide 1
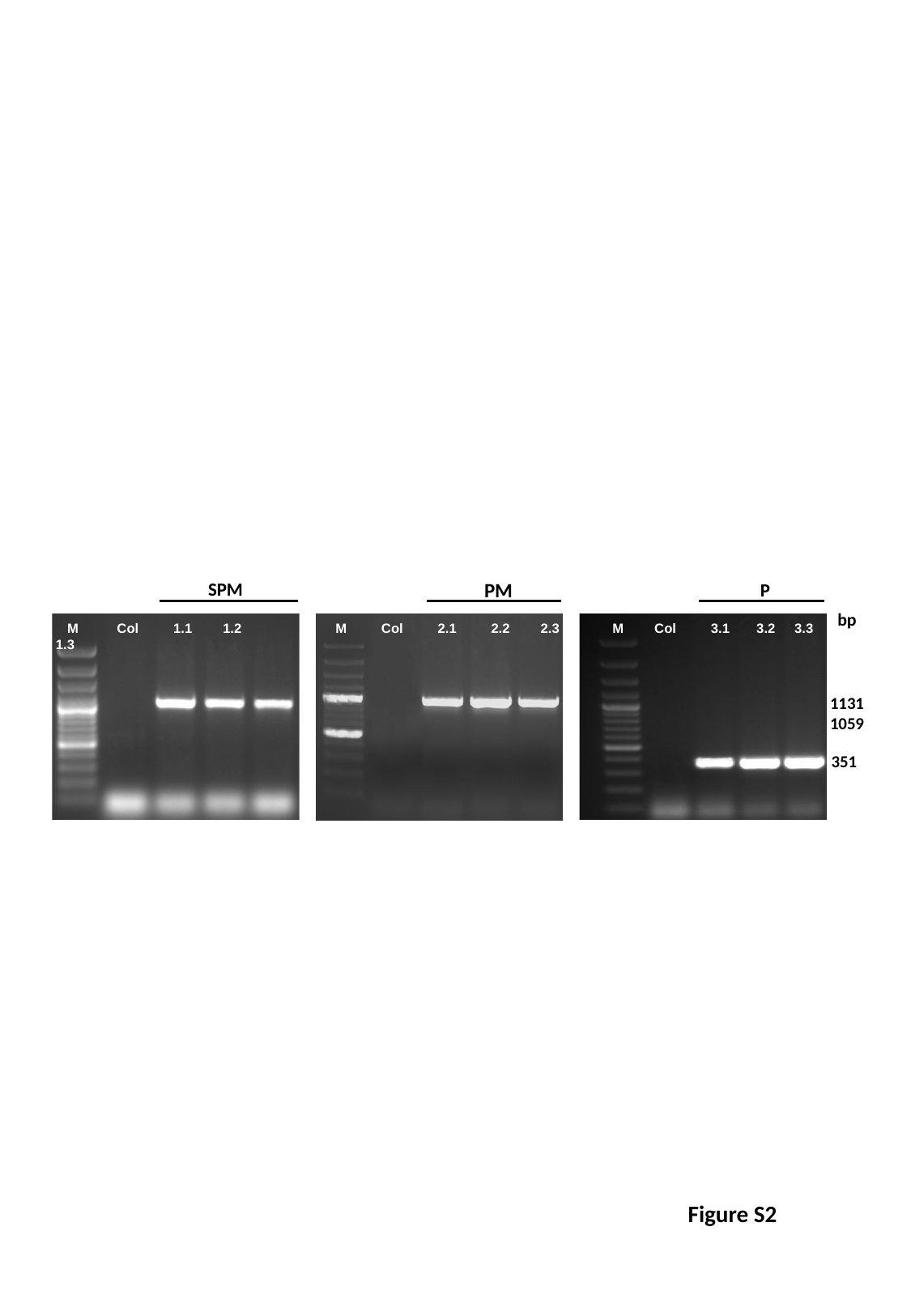

SPM
PM
P
 M Col 1.1 1.2 1.3
 M Col 2.1 2.2 2.3
 M Col 3.1 3.2 3.3
bp
1131
1059
351
Figure S2

Supplement: S2 Fig — Genomic PCR was performed using specific primers described in the Table S2. Transgenic plants were: SPM (lines 1.1, 1.2, 1.3); PM (lines 2.1, 2.2, 2.3); P (lines 3.1, 3.2, 3.3) and non-transformed control (Col). M: 100 bp molecular size marker. Numbers indicate the size of the corresponding amplified fragments. (PPTX) [file pone.0128323.s002.pptx]

## Slide 1
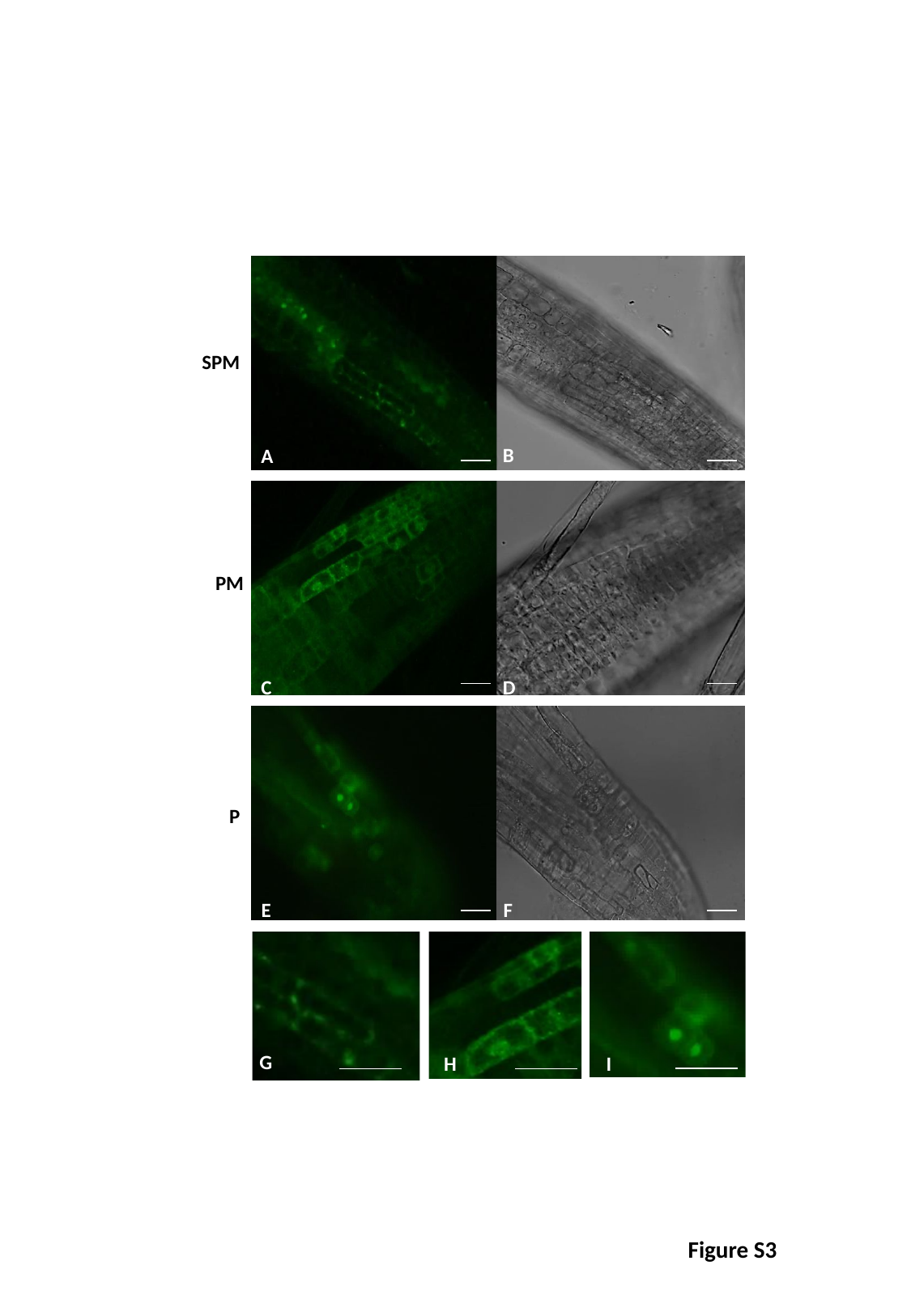

B
A
D
C
E
F
SPM
PM
P
G
I
H
Figure S3

Supplement: S3 Fig — Location of the entire HvPap-1 gene (A and G), the HvPap-1 gene lacking the signal peptide (C and H) and the HvPap-1 pro-peptide sequence (E and I) in mature root cells. Same images were taken under light field conditions (B, D, F). Transgenic plants were: SPM, PM and P lines. Similar images were acquired from three independent transgenic plants. Scale bars: 75 μm (A-I). (PPTX) [file pone.0128323.s003.pptx]
