# Supplementary material for: Inhibitory Properties of Cysteine Protease Pro-Peptides from Barley Confer Resistance to Spider Mite Feeding
Source: PLoS One. 2015 Jun 3;10(6):e0128323. doi: 10.1371/journal.pone.0128323 (PMC4454591; doi:10.1371/journal.pone.0128323)
Supplement: S4 Fig — Adult female mite genes encoding cathepsin L-like (A,B,C,D); cathepsin B-like (E,F); legumain (G,H); cathepsin D-like (I); cystatin (J, K) and thyropin (L) were analysed after 10 days feeding on transformed and non-transformed Arabidopsis lines. Transgenic plants were: SPM plants (lines 1.1, 1.3), PM plants (lines 2.1, 2.3), P plants (lines 3.1, 3.3) and non-transformed control (Col). Data were the mean ± SE of two replicates for each sample. Different letters indicate significant differences (P<0.05, HSD test). (PPTX) [file pone.0128323.s004.pptx]

## Slide 1
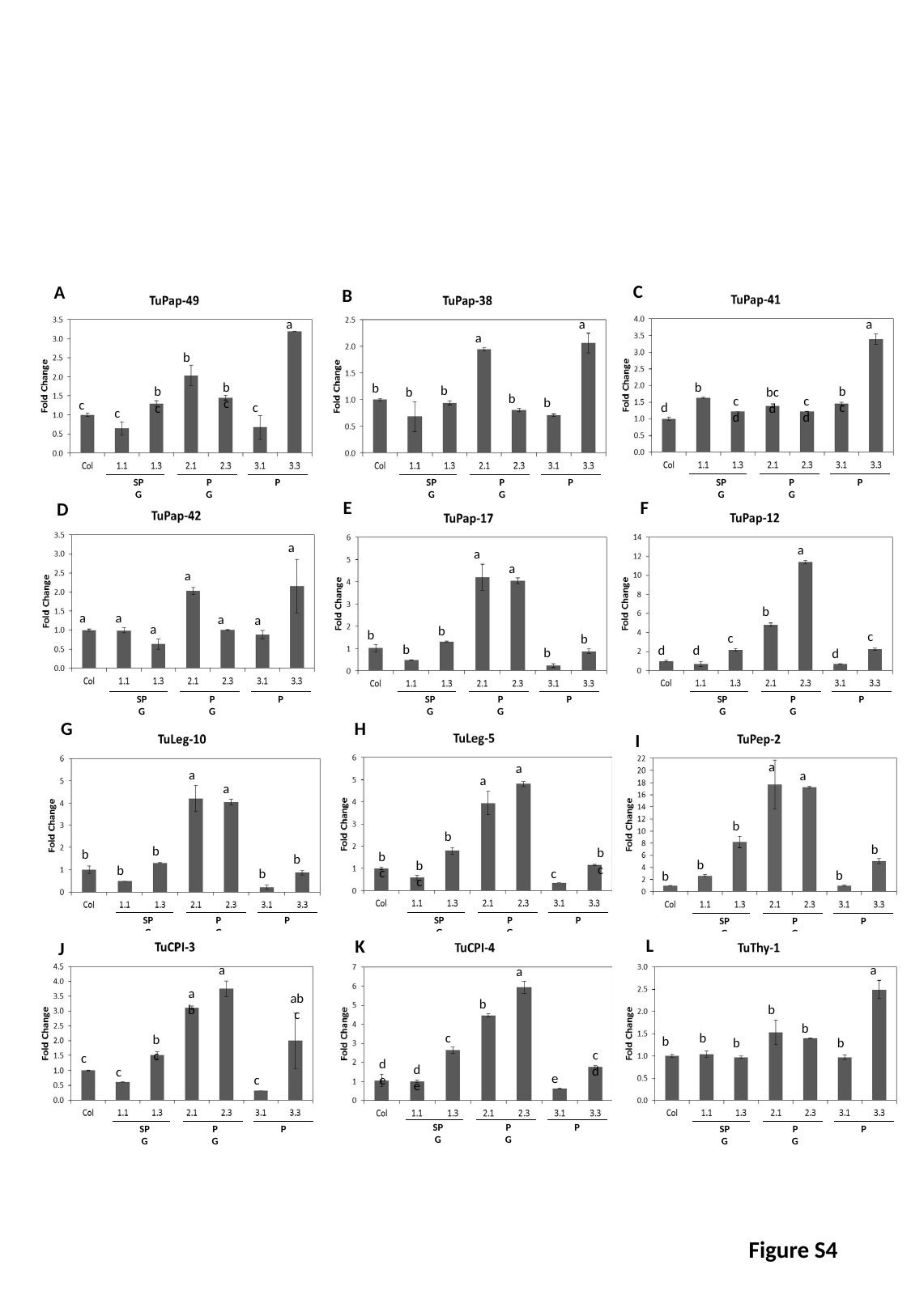

C
a
b
bc
bcd
cd
cd
d
SPG
PG
P
A
a
b
bc
bc
c
c
c
SPG
PG
P
B
a
a
b
b
b
b
b
SPG
PG
P
E
a
a
b
b
b
b
b
SPG
PG
P
F
a
b
c
c
d
d
d
SPG
PG
P
D
a
a
a
a
a
a
a
SPG
PG
P
G
a
a
b
b
b
b
b
SPG
PG
P
H
a
a
b
bc
bc
bc
c
SPG
PG
P
I
a
a
b
b
b
b
b
SPG
PG
P
L
a
b
b
b
b
b
b
SPG
PG
P
K
a
b
c
cd
de
de
e
SPG
PG
P
J
a
ab
abc
bc
c
c
c
SPG
PG
P
Figure S4
