# Supplementary material for: Inhibitory Properties of Cysteine Protease Pro-Peptides from Barley Confer Resistance to Spider Mite Feeding
Source: PLoS One. 2015 Jun 3;10(6):e0128323. doi: 10.1371/journal.pone.0128323 (PMC4454591; doi:10.1371/journal.pone.0128323)
Supplement: S1 Table — (DOCX) [file pone.0128323.s005.docx]

**Table S1.** Effects of the transgenic Arabidopsis lines on *T. urticae* development after feeding bioassay

| Arabidopsis lines | | *T. urticae* developing days Mean ± SE |
| --- | --- | --- |
| CONTROL | Col | 4.88 ± 0.3_a_ |
| SPM | 1.1 | 5.11 ± 0.2_a_ |
|  | 1.3 | 4.79 ± 1^a^ |
| PM | 2.1 | 4.97 ± 0.4_a_ |
|  | 2.3 | 4.94 ± 0.5_a_ |
| P | 3.1 | 4.53 ± 0.6_a_ |
|  | 3.3 | 3.97 ± 0.6_a_ |

Fifteen neonate larvae mites were placed on detached leaves from Arabidopsis lines and every day the emerged nymphs were counted. Results are expressed as mean ± SE of six replicates of independent plants of every transgenic line and non-transformed control. Different letters indicate significant differences (P<0.05, HSD test).
