# Supplementary material for: Inhibitory Properties of Cysteine Protease Pro-Peptides from Barley Confer Resistance to Spider Mite Feeding
Source: PLoS One. 2015 Jun 3;10(6):e0128323. doi: 10.1371/journal.pone.0128323 (PMC4454591; doi:10.1371/journal.pone.0128323)
Supplement: S2 Table — (DOCX) [file pone.0128323.s006.docx]

**Table S2**. Oligonucleotide primers used for conventional PCR of barley *HvPap-1* gene

| CysProt protein (gene) | Gene  fragment | ID^1^ | Oligonucleotide sequences (5’ to 3’) | |
| --- | --- | --- | --- | --- |
|  |  |  | Forward | Reverse |
| Cathepsin F-like  (*HvPap-1*) | SPM | BN000093 | CACCATGGATCATCGCCTCGTGGCC | CTCCTCCTTCGAGGAGTGTGT |
|  | PM |  | CACCATGGCCGCGGGGGACGAGGAG | CTCCTCCTTCGAGGAGTGTGT |
|  | P |  | CACCATGGCCGCGGGGGACGAGGAG | CACCATGGCCGCGGGGGACGAGGAG |

**^1^**NCBI website ([http://www.ncbi.nlm.nih.gov/](http://www.ncbi.nlm.nih.gov/nuccore/BN000093)).
