# Supplementary material for: Inhibitory Properties of Cysteine Protease Pro-Peptides from Barley Confer Resistance to Spider Mite Feeding
Source: PLoS One. 2015 Jun 3;10(6):e0128323. doi: 10.1371/journal.pone.0128323 (PMC4454591; doi:10.1371/journal.pone.0128323)
Supplement: S3 Table — (DOCX) [file pone.0128323.s007.docx]

**Table S3**. Oligonucleotide primers used for RTq-PCR of *Arabidopsis* transgenic lines

| Protein | Gene Name | ID^1^ | Oligonucleotide sequences (5’ to 3’) | |
| --- | --- | --- | --- | --- |
|  |  |  | Forward | Reverse |
| Ubiquitin | AtUbc | At5g25760 | GCTCTTATCAAAGGACCTTCGG | CGAACTTGAGGAGGTTGCAAAG |
| GFP | GFP | BAJ61309 | AGAACGGCATCAAGGTGAAC | TGCTCAGGTAGTGGTTGTCG |

**^1^**TAIR ([http://www.arabidopsis.org/](http://www.ncbi.nlm.nih.gov/nuccore/BN000093)) and NCBI ([http://www.ncbi.nlm.nih.gov/](http://www.ncbi.nlm.nih.gov/nuccore/BN000093)) websites
