# Supplementary material for: Inhibitory Properties of Cysteine Protease Pro-Peptides from Barley Confer Resistance to Spider Mite Feeding
Source: PLoS One. 2015 Jun 3;10(6):e0128323. doi: 10.1371/journal.pone.0128323 (PMC4454591; doi:10.1371/journal.pone.0128323)
Supplement: S4 Table — (DOCX) [file pone.0128323.s008.docx]

**Table S4**. Oligonucleotide primers used for RTq-PCR of *T. urticae* genes

| Protein | Gene Name | ID^1^ | Oligonucleotide sequences (5’to 3’) | |
| --- | --- | --- | --- | --- |
|  |  |  | Forward | Forward |
| Ribosomal | Ribosomal protein 49 | tetur18g03590 | CTTCAAGCGGCATCAGAGC | CGCATCTGACCCTTGAACTTC |
| Cathepsin L-like | TuPap-49 | tetur09g04400 | GCTTTCTCTGCCATTGCTTC | TTTCCATGAGCAATCCATCA |
|  | TuPap-38 | tetur12g01820 | CCATTGCCTCTGTTGAAGGT | CCTTGGTTTCCCTCATCGTA |
|  | TuPap-41 | tetur12g01860 | CCGCTGTTGGTTACGGTACT | CTCTGGCCATTCGGAAGTAA |
|  | TuPap-42 | tetur25g00650 | AAATCAACCGAATGCTCACC | AGCTCCCCAACTGTTCTTGA |
| Cathepsin B-like | TuPap-17 | tetur01g05480 | TCGATGCTCGTGTAGTTTGG | TCAGCAATTGAACCAACAGC |
|  | TuPap-12 | tetur08g05020 | GAACTTGGTGGCCATGCTAT | CAATCGGTGTTCCAGGAGTT |
| Legumain | TuLeg-10 | tetur05g04700 | CAATTCTTTTCGCCCTTTCA | TTGACCCAGCAACCAATACA |
|  | TuLeg-5 | tetur28g01760 | TGAAAATCCCCATCCAGGTA | GTTTTCGGCGTTGACTTCAT |
| Cathepsin D-like | TuPep-2 | tetur14g03010 | CTTTGGGTTCCTTCGTCAAA | CCATTGGGTTTGTAGGTGCT |
| Cystatin | TuCPI-4 | tetur09g03670 | ACCGAAACAGCCAAATCAAC | GCGAGGACCAAAGTGAGTTC |
|  | TuCPI-3 | tetur09g03620 | CCAATTCAGATTCGGTGGAT | GATTTGGCTGTTTCGGTGTT |
| Thyropin | TuThy-1 | tetur09g04580 | AATGCGAGGAAAATGGTCAC | TTCTCACCAATTTGGGTTCC |

**^1^** OrcAE website (<http://bioinformatics.psb.ugent.be/orcae/overview/Tetur>).
